# Tumor Budding, uPA, and PAI-1 in Colorectal Cancer: Update of a Prospective Study

**DOI:** 10.1155/2017/6504960

**Published:** 2017-02-12

**Authors:** Bruno Märkl, Jochen Hardt, Simon Franz, Tina Schaller, Gerhard Schenkirsch, Bernadette Kriening, Reinhard Hoffmann, Stefan Rüth

**Affiliations:** ^1^Institute of Pathology, Klinikum Augsburg, Augsburg, Germany; ^2^Institute of Laboratory Medicine and Microbiology, Klinikum Augsburg, Augsburg, Germany; ^3^Clinical and Population-Based Cancer Registry Augsburg, Augsburg, Germany; ^4^Department of Visceral Surgery, Klinikum Augsburg, Augsburg, Germany

## Abstract

*Aims*. The prognostic role of the proteases uPA and PAI-1, as well as tumor budding, in colon cancer, has been investigated previously.* Methods*. We provide 6-year follow-up data and results of the validation set. The initial test set and validation set consisted of 55 colon cancers and 68 colorectal cancers, respectively. Tissue samples were analyzed for uPA and PAI-1 using a commercially available Enzyme-Linked Immunosorbent Assay (ELISA). Tumor budding was analyzed on cytokeratin-stained slides. Survival analyses were performed using cut-offs that were determined previously.* Results*. uPA was not prognostic for outcome. PAI-1 showed a trend towards reduced cancer specific survival in PAI-1 high-grade cases (68 versus 83 months; *P* = 0.091). The combination of high-grade PAI-1 and tumor budding was associated with significantly reduced cancer specific survival (60 versus 83 months; *P* = 0.021). After pooling the data from both sets, multivariate analyses revealed that the factors pN-stage, V-stage, and a combination of tumor budding and PAI-1 were independently prognostic for the association with distant metastases.* Conclusions*. A synergistic adverse effect of PAI-1 and tumor budding in uni- and multivariable analyses was found. PAI-1 could serve as a target for anticancer therapy.

## 1. Introduction

The role of the microenvironment in cancer has recently gained growing attention. Different conditions can inhibit or enhance tumor growth and dissemination. For example, tumor-associated inflammatory reactions can induce tumor inhibition or promotion. Bioactive molecules released from inflammatory cells to the microenvironment support different hallmark capabilities, including proliferation, cell death limitation, angiogenesis, and matrix modification [1]. Because of its central role in tumor progression, different elements of the microenvironment, such as angiogenesis and the t-cell response, have been identified as interesting targets for anticancer therapy [2]. These general concepts are also valid for colorectal cancer, which is the third leading cause of cancer in the US, with an estimated incidence of approximately 132,000 cases in 2016 [3]. The plasmin/plasminogen system plays an important role in the interaction between the tumor and its matrix. This system consists of the urokinase plasminogen activator (uPA), cell surface receptor urokinase plasminogen activator (uPAR), and its inhibitors plasminogen activator inhibitors 1 and 2 (PAI-1 and PAI-2). UPA and PAI-1 are known to be predictors of aggressive behavior in breast cancer and have been recommended for the diagnosis of node negative breast cancers since 2007. Between 2007 and 2008 our group investigated the potential prognostic value of an ELISA-based uPA and PAI-1 evaluation in colon cancer. Moreover, we assumed that tumor budding, as the histomorphological correlate of epithelial-mesenchymal-transition (EMT), was also associated with the activity of the plasmin/plasminogen system. In this previous study, we found a strong association between elevated uPA levels and tumor budding, as well as high-grade histology. PAI-1 was predictive of distant metastases [4]. We now provide a 6-year follow-up. Additionally, we evaluated another set of cases to validate the findings from the previous study. A subset of rectal cancers was included to estimate whether the findings in these locations were similar to those in colon cancer.

## 2. Materials and Methods

### 2.1. Patients and Specimen Preparation

The test set has been described before. In brief, it consists of 59 colon specimens with 55 cancer lesions that were collected between August 2007 and September 2008. Follow-up data were provided by the clinical and population-based cancer registry of Augsburg, Augsburg, Germany. Additional information was retrieved from the files of the Institute of Pathology and the clinic information system. The physicians that were responsible for further care were contacted to gain information, especially concerning disease control or progression. This study was approved by the ethical committee of the Landesärztekammer Bayern. Informed and written consent was obtained from all patients.

The 68 colorectal cancer cases of the validation set were collected between April and November 2014. This study was approved by the internal review board of the Klinikum Augsburg on the basis of the recommendation by the ethical committee of Landesärztekammer Bayern regarding the previous study. The inclusion criteria for this study were established or suspected colorectal cancer and an elective oncological resection. Emergency surgery or noncurative intentions were criteria for exclusion.

The specimen preparation has been described before. In brief, a 1 cm^3^ tissue block was cut from the invasive tumor region in the fresh tissue immediately after receiving the unfixed specimen. The time between resection and sample collection was less than 30 minutes. In 13 cases from the test set, samples from nonneoplastic regions were also collected. The samples were stored at −20°C for a maximum of 14 days before performing ELISA. Malignancy and the estimation of proportion of the neoplastic cells in 10% steps were confirmed by H&E histology in all samples for the ELISA.

### 2.2. Laboratory Assay

A commercially available ELISA kit for uPA and PAI-1 that is certified for usage in breast cancer was used (Femtelle Test (EF 899), Sekisui Diagnostics, Stamford, CT). The test was conducted according to the protocol recommended by the manufacturer and has been described before [4]. In brief, frozen tissue samples were disrupted by mechanical force under permanent cooling. Tri-Buffer, supplemented with the nonionic detergent Triton X-100, was used to extract the tumor cell cytosol. The suspensions were centrifuged to separate the soluble fractions from the cell debris. The total protein concentrations of the cytosolic fractions were measured. On day 2, a diluted tissue extract was added to antibody-coated microwells and incubated overnight. On day 3, detection antibodies were added and incubated. After another incubation step with the enzyme conjugates, the reactions were stopped with 0.5 M H_2_SO_4_ and the absorption of the solution was measured using a microwell reader at 450 nm. The levels of uPA and PAI-1 were expressed in nanograms per milligram (ng/mg) of tumor protein. Based on the evaluation of the test set [4] the cut-off values for uPA and PAI-1 were ≥4 ng/mg and ≥40 ng/mg, respectively, to discriminate between low and high-grade levels.

### 2.3. Tumor Budding and Immunohistochemistry

Tumor budding was defined as isolated tumor cells or clusters of up to four cells at the invasion front according to Ueno et al. Because of the usage of a microscope with an eyepiece with a field number of 25, the cut-off for high-grade budding was adjusted to ≥30 buds/20-fold magnification in comparison to the original publication [15]. The evaluation was performed on slides that were immunohistochemically stained for pan-cytokeratin to enhance the detectability of single cells. All reactions were performed using a Ventana Benchmark Ultra system. Cell Conditioning Solution (CC1) (Ventana - Roche Diagnostics, Mannheim Germany) was used at the pretreatment step during the processing of all sections. The diagnostic antibody against keratin KL1 (mouse, monoclonal, 1 : 100) was provided by Medac, Wedel, Germany. Independent evaluations were performed by two pathologists (Bruno Märkl and Tina Schaller). In cases of a discrepancy concerning the discrimination between no/low tumor budding and high-grade tumor budding, a consensus decision was made using a double-headed microscope.

Immunohistochemistry for the mismatch repair proteins PMS2 and MSH6 was performed to determine the mismatch repair status in the cases of the validation set where evaluations concerning microsatellite instability (MSI) and/or mismatch repair deficiency (MMRd) have not been performed in the frame of the routine diagnostic. The following diagnostic antibodies were used: PMS2 (Clone EP51, ready to use,) (Clone EP49, ready to use). All reactions were developed using the Ventana Ultravision detection system (Roche Diagnostics, Mannheim, Germany).

### 2.4. Statistical Analysis

The Mann–Whitney Rank Sum test was used to compare numeric values. Correlations were calculated with Pearson Product Correlation. Tabulated data were compared using the *χ*^2^ test or Fisher's Exact test depending on the number of observations. Backward stepwise logistic regression analysis was used to identify independent predictors of metastatic disease. For the survival analysis, Kaplan-Meier Curves were calculated and differences were analyzed with the Log-Rank test. The mean survival times were calculated because the median survival was not reached in most analyses. For the determination of the median follow-up time, the method of Schemper and Smith was used. The Cox regression proportional hazards model was used for the multivariate analysis of cancer specific analysis. The Cohens-Kappa value was calculated to evaluate the interobserver variability during the tumor budding evaluation. All calculations were performed using the Sigma Plot 13.0 software package (Systat, Richmond, VA, USA). *P* values < 0.05 were considered significant.

## 3. Results

### 3.1. Test Set: Patients and Follow-Up

The test set consists of 55 colon cancer cases. Forty-seven of these cases met the criteria for survival analysis. The basic clinicopathological data have been described before and are briefly given in Table 1. The mean and median follow-up times were 75 and 80 months (95% CI: 76.9–83.1).

### 3.2. Test Set: Cancer Specific Survival (CSS)

**Figure 1:**
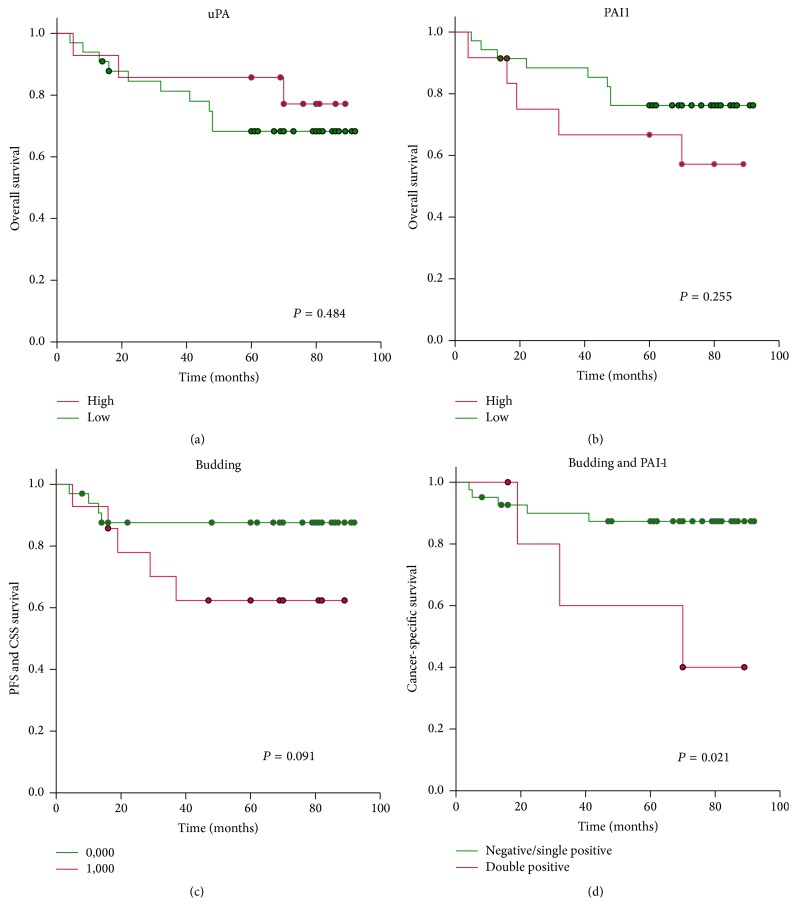
Test set: cancer specific survival of (a) uPA, (b) PAI-1, (c) tumor budding, and (d) a combination of tumor budding and PAI-1 (no or one positive versus double positive). Cut-offs: uPA ≥ 4.0 ng/mg protein; PAI-1 ≥ 40 ng/mg protein; tumor budding ≥ 30 buds/20-fold magnification.

Cancer specific survival was not different in cases with low and high uPA levels with a mean CSS time of 80 months (95% CI: 70–91) versus 76 months (95% CI: 59–94) (*P* = 0.735), respectively. However, a clear trend towards adverse outcome was found for PAI-1. Cases with high PAI-1 levels showed a mean CSS time of 68 months (95% CI: 47–89) compared to low PAI-1 level cases with a CSS time of 83 months (95% CI: 74–93) (*P* = 0.091). High-grade tumor budding was also associated with impaired survival, with a mean CSS time of 71 months (95% CI: 53–89) versus 83 months for low-grade tumor budding (95% CI: 73–33) (*P* = 0.187). Although this difference was not statistically significant, the Kaplan-Meier curves showed a clear discrimination between the two groups. After combining the parameters* tumor budding* and* PAI-1,* the cases with no or only a single positive revealed a significantly higher CCS in comparison to cases that were positive for both. The mean times for negative/single positive versus double positive cases were 83 months (95% CI: 74–91) and 60 months (95% CI: 29–91), respectively (*P* = 0.021). The Kaplan-Meier curves are shown in Figure 1. After performing multivariate analysis including the factors* N-stage*,* vascular invasion*,* PAI-1*,* tumor budding,* and the* combination of PAI1 and tumor budding*, only N-stage was found to be independently prognostic. However, the* combination of PAI-1 and tumor budding* just failed independence, with a P-for-Enter of 0.057.

### 3.3. Validation Set: Patients and Correlations between uPA, PAI-1, and Tumor Budding

The validation set consists of 68 colorectal cancer cases. The clinicopathological data are summarized in Table 2. The two investigators of tumor budding reached a moderate interobserver agreement with a *κ*-value of 0.48.

All samples contained neoplastic cells. The mean percentage of the vital tumor was 640.207 ± 22% (Range 5–90%). There was a weak nonsignificant association between the tumor amount and the level of uPA and no association with PAI-1 (*R* = 0.207, *P* = 0.093; *R* = 0.0594, *P* = 633).

**Figure 2:**
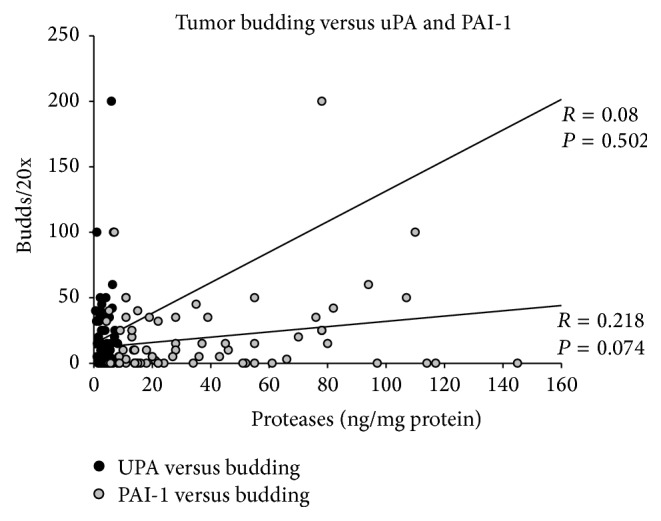
Correlation between tumor budding and uPA and PAI-1.

The mean uPA and PAI-1 levels were 4.0 ± 2.2 and 38.1 ± 33.7 ng/mg protein. Both parameters showed a strong correlation (*R* = 0.709; *P* < 0.001) with each other. However, in this set, tumor budding did not correlate significantly with uPA or PAI-1 (*R* = 0.08 and *R* = 0.218) (Figure 2).

### 3.4. Validation Set: Proteinase Levels and Tumor Budding according to Other Histopathological Factors

**Figure 3:**
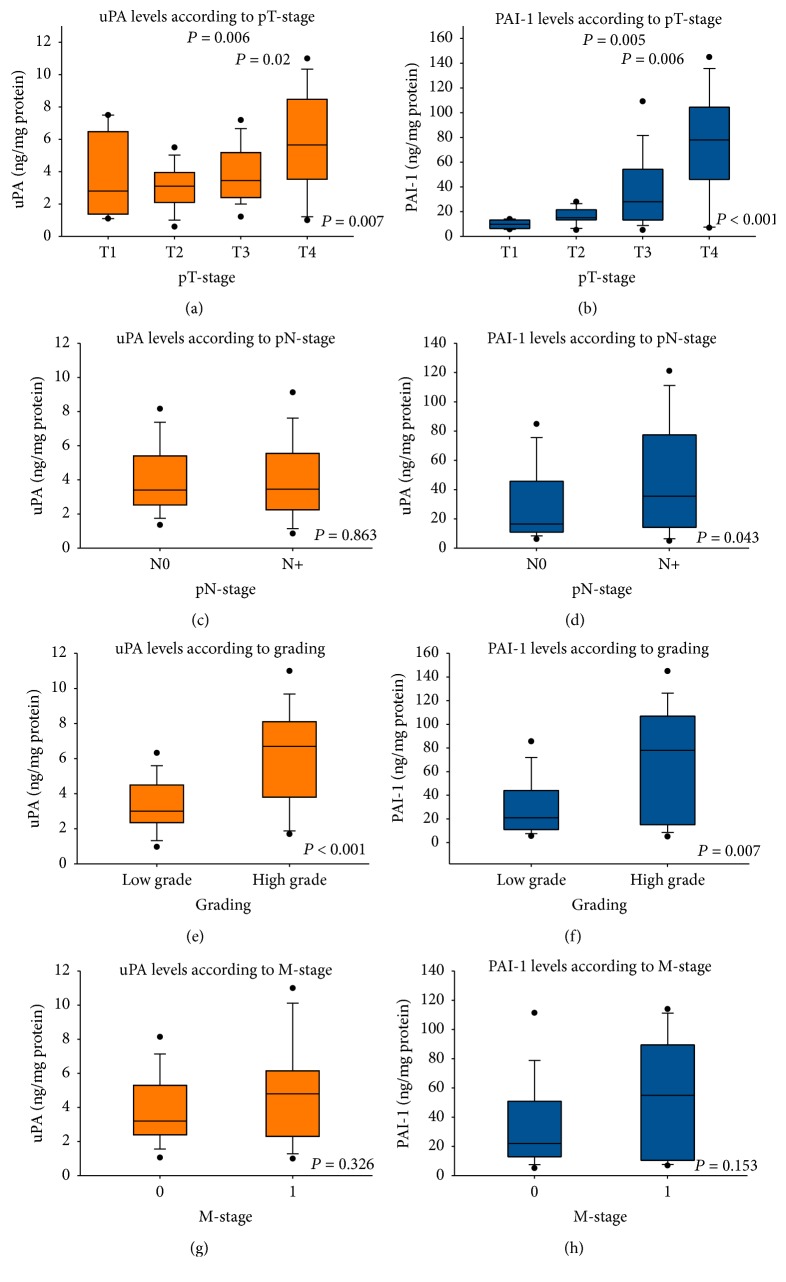
Validation set: mean tissue levels of uPA and PAI-1 according to pT-stage (a and b), pN-stage (c and d), grading (e and f), and M-stage (g and h).

There was no difference in the uPA and PAI-1 levels when comparing cases with and without vascular invasion. Additionally, no difference was found between the uPA levels in node negative or node positive cases (Figure 3(c)). However, there was a significant difference between these subgroups regarding the PAI-1 levels with a median level of 35.5 versus 16.5 ng/mg protein (*P* = 0.043) (Figure 3(d)). The association between nonconventional histological type and high uPA and PAI-1 values is remarkable, in this context (*P* = 0.002; *P* < 0.001). Further significant differences were found for uPA and PAI-1 between the different pT-stages (*P* = 0.007; *P* < 0.001) (Figures 3(a) and 3(b)) and grading (*P* < 0.001; *P* = 0.007) (Figures 3(e) and 3(f)). Additionally, a nonsignificant trend towards higher PAI-1 levels was found in metastatic disease (*P* = 0.153) (Figure 3(h)), whereas the uPA levels showed no significant differences between localized and metastatic disease (Figure 3(g)).

The clinicopathological data, stratified according to the cut-off values for uPA and PAI-1 that were determined during the first evaluation of the test set, are given along with the data for tumor budding in Table 2. In brief, there was a clear trend towards a higher rate of locally advanced cancers (68% versus 89%; *P* = 0.07) and a significantly higher rate of high-grade tumors in the uPA-high group (10% versus 39%; *P* = 0.01). Both parameters were also significantly differentially distributed in the PAI-1 low versus high groups (63% versus 100%; *P* = 0.002 and 50% versus 67%; *P* = 0.01, resp.).

High-grade tumor budding was more highly associated with male gender (*P* = 0.05) and node positivity (50% versus 76%; *P* = 0.091). It was also significantly associated with a left side location (49% versus 65%; *P* = 0.029), venous invasion (10% versus 65%; *P* = 0.022), and metastatic disease (10% versus 47%; *P* = 0.002).

### 3.5. Pooled Data

**Figure 4:**
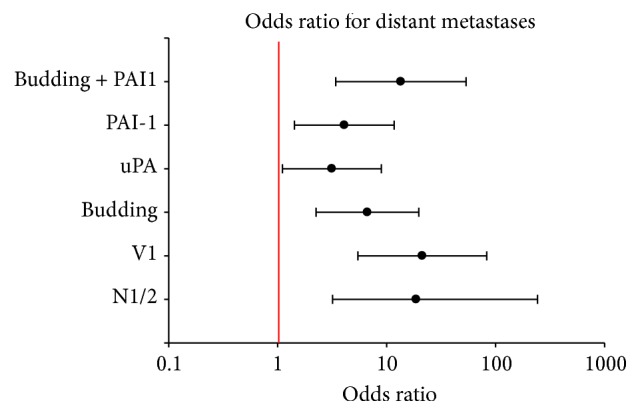
Pooled data: odds ratios of different risk factors for the association with distant metastases. Note the logarithmic scale of the *X*-axis.

After pooling the data from the test and the validation set, the risk for the occurrence of distant metastases was calculated for pT-stage, pN-stage, venous invasion, grading, uPA, PAI-1, tumor budding, and the combination of tumor budding and PAI-1 (Figure 4). Significant prognostic effects were found for pT-stage, pN-stage, venous invasion, PAI-1, tumor budding, and the combination of tumor budding and PAI-1. The corresponding odds ratios were inf., 30.8, 21.3, 4.1, 6.7 and 13.5, respectively. UPA just failed to meet significance with *P* = 0.051 (OR: 3.5). The multivariate analyses revealed that the factors pN-stage, venous invasion, and combination of tumor budding and PAI-1 were independently prognostic for the occurrence of distant metastases.

## 4. Discussion

Here, we provide follow-up data from a prospective study that was initially performed in 2007/2008 to investigate the role of the serine proteases uPA and PAI-1 in tumor budding in colon cancer [4]. Moreover, we present the initial data from a recently performed validation study. The survival analysis of the test set revealed a nonsignificant trend towards an adverse outcome for patients with high PAI-1 tissue concentrations and high-grade tumor budding. The fact that a combination of both factors leads to a significant discrimination regarding cancer specific survival can be seen as an indication that both factors interact and enhance tumor cell migration and tumor progression. Tumor budding is characterized by the loss of E-cadherin, a mesenchymal phenotype, and detachment from neoplastic glands [16–20]. Other authors have described similar connections between the function of the plasmin system and tumor budding. Recently, Sánchez-Tilló et al. reported that ZEP1 regulates uPA and PAI and is expressed in dedifferentiated cells at the invasion front that have lost E-cadherin [13]. Minoo et al. found that immunohistochemical overexpression of uPA and the loss of E-cadherin and APAF-1 are predictive of the infiltrating tumor border [7]. Hiendlmeyer et al. found that *β*-catenin upregulates uPA expression. The evaluation of our test set revealed strong correlations between tumor budding and uPA and PAI-1 levels [22]. However, in our validation set, we only found a weak correlation between budding and PAI-1 with marginal significance. The median PAI-1 and uPA levels were evaluated in cases with high-grade budding. An interesting finding is that uPA and PAI-1 levels were strongly associated with the histological type in the validation set. Specifically, the mucinous type was significantly more often found in the uPA- and PAI-1-high groups. Reevaluating the test set revealed a trend (*P* = 0.167) in this direction.

In our initial study, we showed an association between the tissue levels of uPA and PAI-1 and aggressive histopathological features of colon cancers. The recently finished validation study confirmed the role of these proteases as negative prognostic markers in colon cancers. This was true when the mean values were compared and also when the collection was stratified according to previously determined cut-off values. However, it has to be stated that the combinations of factors that were associated with each other were not completely identical (Table 3), which is very likely influenced by the relatively small number of cases for these studies, including all stages of cancers, which is a clear limitation. Another limitation is that the two studies are not optimally balanced. The validation study included proportions of right sided, nodal positive cancers, as well more cases with vascular invasion and distant metastases, which may have also contributed to the instability found in the results. Nevertheless, whenever significant differences were found, they indicated an association of the proteases with aggressive behavior. This was especially true for PAI-1 and tumor budding.

A multivariable analysis performed after pooling the data from both sets revealed that pN-stage, V-stage, tumor budding, and PAI-1 were independently associated with distant metastases. The prognostic role of PAI-1 in colorectal cancers has been evaluated by several other groups, and the overwhelming majority of studies confirmed that PAI-1 is an adverse factor [4]. An overview of the more recently published literature is given in Table 4 [6–14]. Wang et al. performed a meta-analysis to evaluate the influence of the PAI-1 4G/5G polymorphism. Individuals with a 4G/4G genotype have increased PAI-1 plasma levels and show an increased susceptibility to colorectal and endometrial cancer [23]. Iacoviello et al. also found increased plasma levels of PAI-1 in patients with colon cancers compared to controls in a large retrospective analysis [12]. Experimental data came from Hogan et al. and Chen et al., who show that cancer cell functions are influenced by PAI-1 [10, 14]. Moreover, Chen et al. demonstrated compromised tumor growth caused by the silencing of PAI-1.

In the analyses of the test and validation sets, UPA was found to be inferior concerning its prognostic significance in comparison to PAI-1 and tumor budding. Kushlinskii et al. also found PAI-1, but not uPA, to be prognostic in colorectal cancer [9]. However, this result is in contrast to those of other studies that reported strong correlations with aggressive behaviors, including impaired survival rates [7, 24–29]. Most of these studies were performed using immunohistochemistry, which could be a technical explanation for this discrepancy. Ganesh et al. and Herszènyi et al. used ELISA and also reported impressive survival differences [25, 30]. The main difference between their investigations and ours is that they used a different ELISA. However, it seems unlikely that this can fully explain this strong discrepancy. It remains unclear why we could not confirm the data of these authors.

Tumor budding is gaining recognition as a promising and robust prognostic factor in colorectal cancer. It is especially interesting for the evaluation of malignant polyps in the preoperative setting and in stage II cancers [31]. Its prognostic relevance in the whole gastrointestinal tract has been investigated in a large number of studies [31, 32]. However, there are still two main issues with these factors that remain unsolved. The most important of these issues is the fact that an accepted consensus on how tumor budding should be evaluated is lacking. The other point is the relatively weak interobserver agreement, at least in some of the publications [32]. Puppa et al. found a fair agreement between the international participants of their study. Cytokeratin immunohistochemistry improved the agreement [33]. In our study, we also achieved only moderate interobserver agreement, with a *κ*-value of 0.48. It is astonishing that, despite these limitations, tumor budding has been demonstrated to be a robust adverse factor many times. An increased risk for lymph node metastases in pT-1 cancers has been confirmed in systematic reviews and meta-analyses [34–36]. Several authors and societies recommend the evaluation of tumor budding [37–40], which underlines its importance. In our study, we also found an association between high-grade tumor budding and other adverse factors and outcomes.

Despite investigating another set of 68 cases in addition to the initial collection, this study is still hampered by a relatively low case number. Nevertheless, the data of this study with a prospective design can serve as a basis for further investigations. We found a synergistic adverse effect of PAI-1 and tumor budding in uni- and multivariable analyses. This supports the thesis that stromal degradation facilitates the migration of detached tumor cells. Because of the association with the histological type further investigations should focus on this by comparing collectives with identical UICC stages, MSI status, and/or locations. Because PAI-1 inhibitors are already available, additional investigations should address the question of whether PAI-1 could serve as a target for anticancer therapy [41].

## Figures and Tables

**Table 1:** Clinicopathologic data: test set.

	Malignant (*n* = 55)	Nonmalignant (*n* = 4)
Mean age ± SD	71 ± 13	68 ± 8
Gender (m : f)	0.57 : 1	0.33 : 1
Right colon	19	3
Left colon	36	1
Conventional histological type	48	/
Mucinous type	4	/
Medullary type	1	/
Micropapillary type	2	/
Infiltrative invasion type^*∗*^	4	/
pT 1/2	16	/
pT 3/4	39	/
Low/moderate grade	39	/
High grade	16	/
Node positive	21	/
Lymphatic invasion	4	/
Venous invasion	6	/
Perineural invasion	3	/
Strong chronic inflammation	11	/
Metastatic disease	7	/
Mean LN, number ± SD	33 ± 18	19 ± 6

^*∗*^Infiltrative type according to Jass. SD, standard deviation; LN, lymph nodes.

**Table 2:** Clinicopathological data: validation set.

	Complete collection (*n* = 68)	uPA low (*n* = 40)	uPA high (*n* = 28)	*P* value	PAI-1 low (*n* = 44)	PAI-1 high (*n* = 24)	*P* value	Budding low (*n* = 51)	Budding high (*n* = 17)	*P* value
Mean age ± SD	68 ± 11	68 ± 10	67 ± 12	*1.0*	68 ± 11	67 ± 12	*0.753*	69 ± 11	64 ± 11	*0.091*
Gender m : f	1 : 0.9	1 : 0.74	1 : 1.15	*0.514*	1 : 0.7	1 : 1.4	*0.262*	1 : 1.2	1 : 0.3	*0.05*
Right colon	32 (47%)	17 (43%)	15 (54%)		21 (48%)	11 (46%)		26 (51%)	6 (35%)	
Left colon	18 (26%)	11 (28%)	7 (25%)		9 (20%)	9 (38%)		16 (31%)	2 (12%)	
Total colon	3 (4%)	1 (3%)	2 (7%)	0.462^*∗*^	1 (2%)	2 (8%)	0.103^*∗*^	1 (2%)	2 (12%)	0.029^*∗*^
Rectum	15 (22%)	11 (28%)	4 (14%)	0.319^#^	13 (30%)	2 (8%)	0.087^#^	8 (16%)	7 (41%)	0.043^#^
Conventional histological type	56 (82%)	38 (95%)	18 (64%)		42 (95%)	14 (58%)		42 (82%)	14 (82%)	
Mucinous type	8 (12%)	1 (3%)	7 (25%)		1 (2%)	7 (29%)		6 (12%)	2 (12%)	
Medullary type	1 (1%)	0 (0%)	1 (4%)		0 (0%)	1 (4%)		1 (2%)	0 (0%)	
Micropapillary type	3 (4%)	1 (3%)	2 (7%)	0.002^*∗∗*^	1 (2%)	2 (8%)	<0.001^*∗∗*^	2 (4%)	1 (6%)	1.0^*∗∗*^
Infiltrative type^‡^	6 (9%)	6 (15%)	0 (0%)	*0.164*	6 (14%)	0 (0%)	*0.083*	3 (6%)	3 (18%)	*0.160*
pT 1/2	16 (24%)	13 (33%)	3 (11%)		16 (36%)	0 (0%)		14 (27%)	2 (12%)	
pT 3/4	52 (76%)	27 (68%)	25 (89%)	*0.073*	28 (64%)	24 (100%)	*0.002*	37 (73%)	15 (88%)	*0.322*
Grading: low grade	53 (78%)	36 (90%)	17 (61%)		39 (89%)	14 (58%)		41 (80%)	12 (71%)	
Grading: high grade	15 (22%)	4 (10%)	11 (39%)	*0.01*	5 (11%)	10 (42%)	*0.01*	10 (20%)	5 (29%)	*0.501*
Node positive	38 (56%)	21 (53%)	17 (61%)	*0.404*	22 (50%)	16 (67%)	*0.286*	25 (49%)	13 (76%)	*0.091*
Lymphatic invasion	15 (22%)	10 (25%)	5 (18%)	*0.563*	9 (20%)	6 (25%)	*0.762*	8 (16%)	7 (41%)	*0.043*
Venous invasion	11 (16%)	6 (15%)	5 (18%)	*0.319*	6 (14%)	5 (21%)	*0.500*	5 (10%)	6 (35%)	*0.022*
Perineural invasion	10 (15%)	5 (13%)	5 (18%)	*0.730*	7 (16%)	3 (13%)	*1.000*	6 (12%)	4 (24%)	*0.254*
Strong chronic inflammation	13 (19%)	6 (15%)	7 (25%)	*0.357*	10 (23%)	3 (13%)	*0.355*	13 (25%)	0 (0%)	*0.028*
MSI/MMRd	9 (13%)	3 (8%)	6 (21%)	*0.146*	5 (11%)	4 (17%)	*0.710*	9 (18%)	0 (0%)	*0.099*
Metastatic disease	13 (19%)	5 (13%)	8 (29%)	*0.124*	6 (14%)	7 (29%)	*0.195*	5 (10%)	8 (47%)	*0.002*
Mean LN, number ± SD	43 ± 17	44 ± 20	42 ± 12	*0.837*	42 ± 19	45 ± 13	*0.068*	43 ± 18	42 ± 16	*0.949*
Neoadjuvant therapy	7(47%)^*ǂ*^	4 (36%)	3 (75%)	0.282^*ǂ*^	6 (46%)	1 (50%)	1.000^*ǂ*^	3 (38%)	4 (57%)	0.619^*ǂ*^

SD, standard deviation; LN, lymph nodes. ^*∗*^Calculation includes all locations (right, left, total, and rectum). ^#^Rectal versus nonrectal cancers. ^*∗∗*^Conventional versus group of nonconventional types. ^‡^According to Jass [5]. ^*ǂ*^Basis is the number of rectal cancers.

**Table 3:** Comparison of the results: test set versus validation set.

	uPA			PAI-1			Budding	
	Test set mean values	Val. set mean values	Val.-set cut-off	Test set mean values	Val. set mean values	Val. set cut-off	Test set mean values	Val. set cut-off
Grading	***Y***	***Y***	***Y***	*N*	***Y***	***Y***	***Y***	*N*
T-stage	*N*	***Y***	**T**	*N*	***Y***	***Y***	*N*	*N*
N-stage	*N*	*N*	*N*	*N*	***Y***	*N*	***Y***	**T**
V-stage	*N*	*N*	*N*	***Y***	**T**	*N*	*N*	***Y***
M-stage	Y^*∗*^	**T**	**T**	Y^*∗*^	**T**	***Y***	Y^*∗*^	***Y***

Y, statistically prognostic; N, not statistically prognostic; T, trend towards prognostic relevance; bold and italic fonts, concordance between test and validation set; bold italic font, discordance between test and validation set. ^*∗*^Evaluation according to cut-off stratification.

**Table 4:** Previous studies investigating the prognostic role of uPA and PAI-1 in colorectal cancer.

References	Year	*n*	Specimen	Protease	Method	Results
Mutoh et al. [6]	2008	25	Mice experimental	PAI-1 and PAI-1 inhibitors SK116 and 215	ELISA, RT-PCR, polyp formation, tissue	PAI-1 Blockers suppress polyp formation
Minoo et al. [7]	2009	975	Colorectal	uPA, uPAR	IHC, microarray	uPA but not uPAR is independently prognostic
Yamada et al. [8]	2010	100	Colorectal	PAI-1	ELISA, plasma	Predictive of postoperative recurrence
Kushlinskii et al. [9]	2013	166	Colorectal	uPA, PAI-1, tPA	ELISA, plasma	PAI-1 prognostic but only in univariate analysis
Hogan et al. [10]	2013		Colon cancer and mesenchymal stem cells	PAI-1	Cell cultures	PAI-1 is secreted by MSC
Kim et al. [11]	2013	3136	Colorectal	PAI-1	ELISA, plasma	No independent association between PAI-1 and polyp-formation; univariable weakly significant
Iacoviello et al. [12]	2013	850	Colorectal	PAI-1	ELISA, plasma	Risk factor for colorectal cancer
Sánchez-Tilló [13]	2013		Colorectal		Cell cultures	ZEB1 regulates uPA and PAI1 and promotes invasiveness
Chen et al. [14]	2015	108	Colorectal	PAI-1	ELISA, plasma, and cell cultures	Silencing of PAI-1 suppresses CR cancer progression

ELISA, Enzyme-Linked Immunosorbent Assay; RT-PCR, reverse transcription polymerase chain reaction; IHC, immunohistochemistry; MSC, mesenchymal stem cells; CR, colorectal. Note: literature from 1993–2008 is given in [4].
